# Menstrual Hygiene Practices in Indian Tribal Females: A Systematic Review and Meta-Analysis

**DOI:** 10.7759/cureus.42216

**Published:** 2023-07-20

**Authors:** Swati Mittal, Swati Priya, Rajan Kumar, Bhavna Verma, Anamika Meena

**Affiliations:** 1 Physiology, All India Institute of Medical Sciences Deoghar, Deoghar, IND; 2 Obstetrics and Gynecology, All India Institute of Medical Sciences Deoghar, Deoghar, IND; 3 Pediatrics, All India Institute of Medical Sciences Deoghar, Deoghar, IND; 4 Radiodiagnosis, All India Institute of Medical Sciences Patna, Patna, IND

**Keywords:** dustbin disposal, indian tribal females, menstrual hygiene, menstrual materials, sanitary pads

## Abstract

India is native to many tribal communities: Bharia (Madhya Pradesh), Bihl (Rajasthan), Santhal (Bihar, Jharkhand), Bodo (Assam, West Bengal), and many more. They reside in isolated geographical regions, which poses challenges in reaching out to them. In addition, they still have firm beliefs and taboos regarding menstruation. Knowledge about menstrual health and hygiene is one of the most important aspects of tribal health. Therefore, it is important to synthesize the results of menstrual hygiene data from the Indian tribal population. We have calculated the pooled prevalence of sanitary pad use, dustbin disposal, and hygienic reuse of menstrual materials.

Online databases, namely PubMed, Cochrane Central, CINAHL, Pan African Journals, EBSCO, and Google Scholar, were searched. After the removal of duplicates, a quality check, and screening of cross-references, 19 articles were selected for final review. Statistical analysis was done by Revman 5.4 and STATA 17.0. A p-value of <0.05 was considered statistically significant. PRISMA guidelines were followed. The protocol registration number was CRD42022331376. This is a non-funded article.

The pooled prevalence of sanitary pad use in Indian tribal females was 2% (95% CI 1 to 3). The pooled prevalence of dustbin disposal of menstrual material was 1% (95% CI: 0.00 to 0.02). The pooled prevalence of hygienic reuse of menstrual materials was 1%.

Sanitary menstrual hygiene practices are very less prevalent in Indian tribal females. Awareness programs and tribal health policies need to be accelerated for the promotion of menstrual hygiene. Also, literature on the use, disposal, and storage of menstrual adsorbents is scarce in Indian tribes. Health research in this area needs to be emphasized.

## Introduction and background

Menstrual hygiene is an integral part of tribal health [1]. In India, menstruation is regarded as a social and cultural taboo [2]. Lack of knowledge and social support cause anxiety and fear, leading to unpreparedness for menstruation and unsafe menstrual practices in young females [3].

Sanitary pads and sanitary napkins have been made available under many government programs for promoting menstrual hygiene [4]. Apart from the type of adsorbent used, the method of use, disposal, storage, and reuse practices are equally important [5]. Hulland KRS et al. noted that sanitation behaviors that were most restricted, namely menstruation, were the most stressful. This is remarkable in young adolescents and newly married women living in their in-laws’ households, where they face social restrictions surrounding menstruation and all sanitation-related behaviors such as restricted water access, taboos related to cooking, or religious practices during their periods [6].

India is the homeland of a diversity of tribes distributed widely across the country. They usually reside in hilly terrain and transit-restricted areas that are difficult to reach [7]. Every tribal community is unique with respect to practices, beliefs, and customs [8]. Previous literature shows varied practices related to menstrual management are prevalent differently in different tribes. Further, isolation, religious and lifestyle restrictions, and taboos on the consumption of foods such as rice, curd, milk, lassi, potatoes, and onion sugarcane during the menstrual period are imposed [9-11].

Hence, this study was undertaken to determine the pooled prevalence of usage, dustbin disposal, and sun-drying practices of menstrual materials among Indian tribal females.

## Review

Methods

Search Strategy, Quality Appraisal, and Study Selection

Detailed strategic searches were undertaken on the websites of PubMed/Medline, Cochrane Central, CINAHL, Pan African Journals, and EBSCO. The first 10 pages of Google Scholar were also screened. The articles published in the last 10 years (2013-2023) were included. The search terms used were “sanitary pads,” “sanitary napkins,” “menstrual hygiene material,” “menstrual adsorbent,” “menstrual hygiene,” “menstrual hygiene practices”, AND “tribal females,” “tribal girls,” “tribal adolescents”, AND “India,” OR “Indian.” Initial searches were undertaken by two investigators (R.K. and S.M.) independently. The differences were discussed and resolved by another investigator (S.P.). Additional searches included cross-references to included articles and a bibliography of review articles. Duplicates and articles with missing main variable data were excluded.

Quality appraisal of the articles was done by the Joanna Briggs Institute’s (JBI) Meta-Analysis of Statistics Assessment and Review. Instruments and articles with more than a 50% score were selected. Finally, 19 articles were included in the final review and meta-analysis. Preferred Reporting Items for Systematic Reviews and Meta-analysis (PRISMA) guidelines were followed in the manuscript development process. The protocol was registered in the PROSPERO Register of systematic reviews (registration number CRD42022331376).

Inclusion/Exclusion Criteria

All descriptive studies with data on the prevalence of sanitary pad use published in the past 10 years were included. The RCTs comparing menstrual hygiene materials (MHMs) with prevalence data on sanitary pad use were also included.

The trials with missing data on menstrual adsorbent use and descriptive studies on the physiology of menstruation and other menstrual concerns (like heavy menstrual bleeding, polycystic ovaries, dysmenorrhea, adenomyosis, and fibroid) were excluded. The abstracts for which full text could not be sought despite contacting the authors thrice were also excluded.

Statistical Analysis

The prevalence of sanitary pad use in the study population was taken as the primary study variable. The prevalence of sanitary pad/napkins disposal and the prevalence of drying sanitary napkins before reuse were also extracted from the included articles and synthesized. The statistical analysis software used was RevMan 5.4 and STATA 17.0. The pooled prevalence of all three variables was calculated using a fixed and random effects model. The mean difference was used as the effect measure for the presentation of results. A p-value of less than 0.05 was considered statistically significant.

Results

Study Selection and Quality Appraisal

After identification, 111 articles were taken for full-text screening. Fifty-two irrelevant articles were removed as they were not related to the topic. Three articles were undertaken outside India, and 28 articles were not conducted with tribal females. Excluded studies were conducted by Ganguly L [12], which compared sanitation and infrastructure facilities in tribal and non-tribal females, and Tzeghai GE [13], which reported a novel method of menstrual adsorbent quality assessment. Further, Acthunan K [14] noted the good performance of the novel reusable banana fiber pad. These articles did not mention complete descriptive data, so they were excluded.

**Figure 1:**
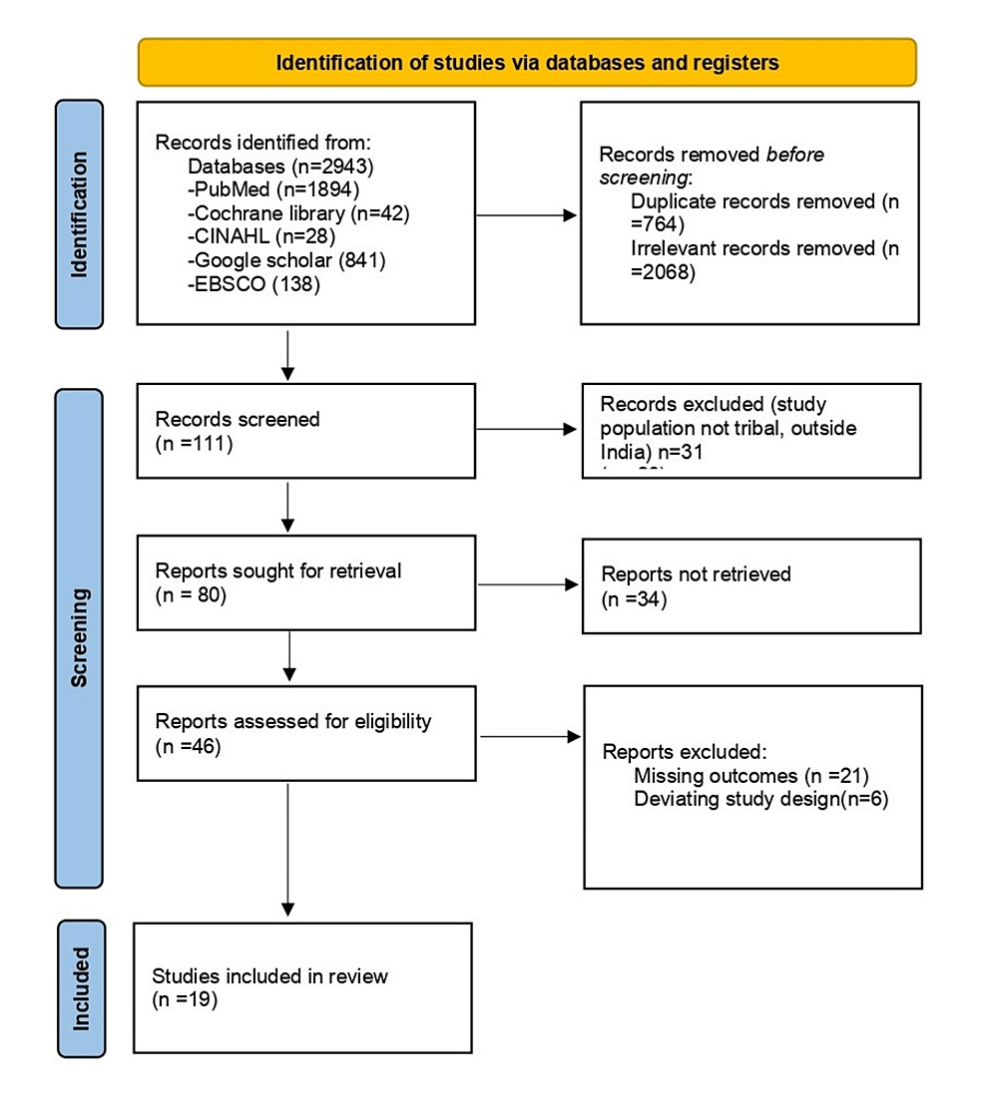
PRISMA flowchart for study selection PRISMA: Preferred Reporting Items for Systematic Reviews and Meta-Analysis

A quality appraisal was done for 47 articles, out of which 19 did not report the outcome variable, and 11 were experimental studies with insufficient descriptive data. The articles that scored four and above out of eight points by the JBI assessment tool [15] were included in the resulting synthesis. Two articles were also identified by a cross-reference search of review articles and included (Figure 1).

Finally, 19 articles were included for meta-analysis. 

Characteristics of the Included Studies

After the study selection process, 19 studies of 8062 participants published from 2013 to 2023 in Indian tribal females were included in the final meta-analysis. All 19 studies reported data on the prevalence of the use of sanitary pads in the study population. The prevalence of dustbin disposal of sanitary pads was reported in nine articles, and the prevalence of drying reusable cloth pads under sunlight was mentioned in seven articles. The study characteristics are tabulated in Table 1.

**Table 1:** Study characteristics of included studies *MHM: Menstrual Hygiene Material, **UTI: Urinary Tract Infection

Author, Year	Sample Size	Study Design	Place of Study	Outcome
Kumar D et al., 2021 [16]	22	Pilot survey	Bharia women, Chhindwara, Madhya Pradesh	75% of adolescents and 71.4% of adult women cleaned their genitals with soap and water.
Clarke KR et al., 2019 [17]	3324	Cross-sectional study	West Singhbhum, Jharkhand	48.4% of women used sanitary pads.
Borkar SK et al., 2022 [18]	272	Cross-sectional study	Nagpur, Maharashtra	38.97% of girls cleaned their genitalia with soap and water.
Sridhar D et al., 2017 [19]	425	Cross-sectional study	Achampet mandal, Telangana	55.1% of menstrual hygiene was non-sanitary. No literacy in participants and their mothers. Education and awareness play a key role in maintaining menstrual hygiene.
Udayar SE et al., 2016 [20]	239	Community based Cross-sectional study	Kuppum Mandal, Andhra Pradesh	82.9% of girls used water only. 17.1% of girls used soap and water for cleaning external genitalia.
Shah SP et al., 2013 [21]	164	Community based study	South Gujarat	Falalin cloths were readily available, easy to use, and cheaper than sanitary pads.
Pal D et al., 2022 [22]	106	Descriptive study	Behu tribe Madhya Pradesh	99% of washed genitalia before and after changing MHM*
Sawa T et al., 2022 [23]	20	Cross-sectional survey	Jharkhand	35% of women used cloth during menses.
Kakwani J et al., 2021 [24]	76	Cross-sectional survey	Udaipur, Rajasthan	None used sanitary pads. 100% unsafe disposal by throwing pads in village ponds.
Kakeri M et al., 2018 [25]	227	Cross-sectional study	Palghar, Maharashtra	17.1% of females used an antiseptic solution along with water for cleaning the genitalia.
Ramya S et al., 2020 [26]	400	Cross-sectional study	Salem, Tamil Nadu	54% of females had good menstrual hygiene practice. Reuse of pads (100%). Clean with soap and water (100%).
Dey J et al., 2020 [27]	60	Cross-sectional study	Medinipur, West Bengal	78.3% of tribal females had no concept of the menstrual cycle before menses.
Verma A et al., 2021 [28]	200	Community-based cross-sectional study	Udaipur, Rajasthan	Frequency of change (40%) thrice daily. 86% of females cleaned their genitalia with soap and water.
Das P et al., 2015 [29]	486	Cross-sectional study	Bhubaneswar, Odisha	Women using reusable cloths were twice as likely to get UTI**
Mishra P et al., 2020 [30]	740	Cross-sectional questionnaire-based study	Kandhamal, Odisha	38% of girls used sanitary pads during menstruation. 22% of girls changed pads.
Mahapatra T et al., 2023 [31]	450	Community-based cross-sectional study	Balasore, Odisha	61.7% of girls used sanitary pads.
Birje S et al., 2022 [32]	45	Focused Group Discussion questionnaire	Palghar, Maharashtra	Reusable cloth is kept in a room corner that no one can see and reused for one year.
Kumari S et al., 2021 [33]	150	Community-based cross-sectional study	Khunti, Jharkhand	None used sanitary pads. 48.67% of girls were aware of menstruation before menarche.
Vayeda M et al., 2021 [34]	550	Implementation capacity building interventional study	Narmada, Gujarat	Pad change frequency: Thrice a day 11.4% of females routinely burned used MHM* in backyard with dried waste.

Results of Synthesis

**Figure 2:**
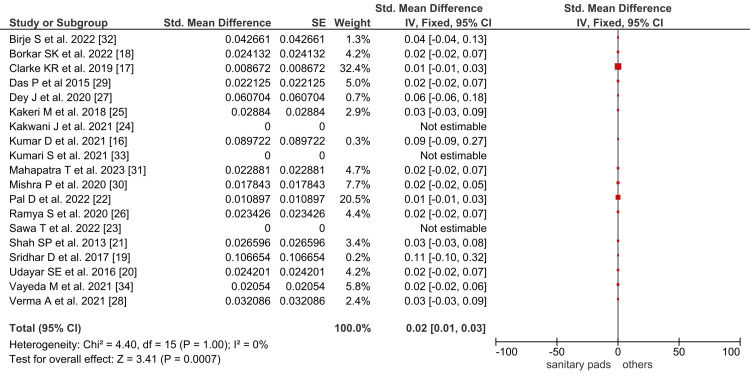
Forest plot showing the pooled prevalence of sanitary pad use in Indian tribal females

The data on menstrual hygiene from the included studies on tribal Indian females were synthesized. The prevalence of sanitary pad usage, dustbin disposal, and sunlight drying was plotted using Forrest plots using Rev Man and STATA software. The pooled prevalence of sanitary pad use in Indian tribal females was 2% (95%, CI: 1 to 3), as shown in Figure 2.

**Figure 3:**
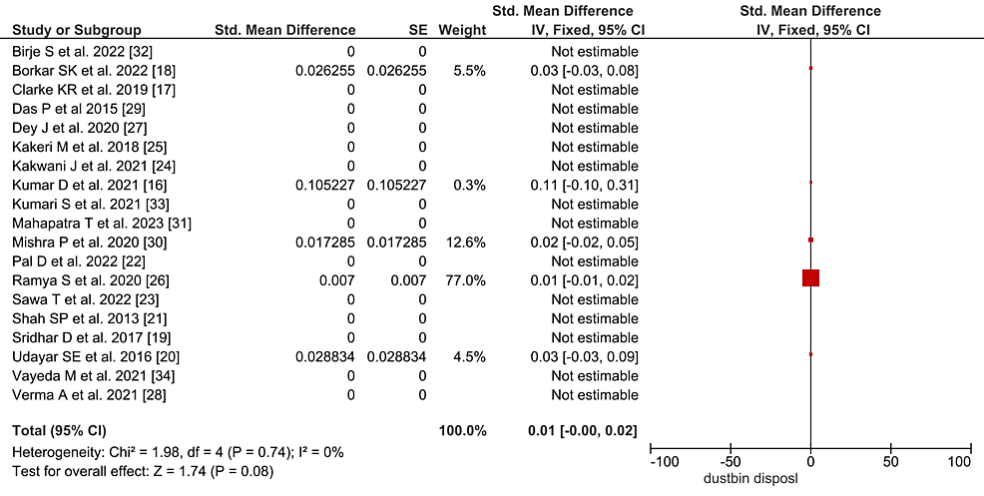
Forest plot showing the pooled prevalence of dustbin disposal of menstrual materials in Indian tribal females

The pooled prevalence of dustbin disposal of sanitary pads in Indian tribal females was 1% (95%, CI: 0.00 to 0.02), as noted in Figure 3.

**Figure 4:**
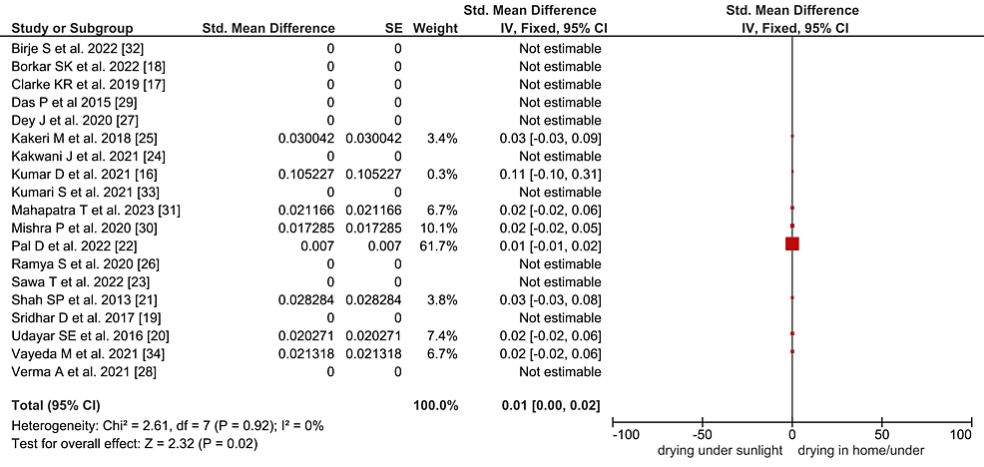
Forest plot showing the pooled prevalence of sunlight drying menstrual materials in Indian tribal females

The pooled prevalence of drying under sunlight of reusable cloth pads was 1% (95% CI: 0.00 to 0.02), as depicted in Figure 4.

Risk of Bias

All included articles reported data on the prevalence of the use of sanitary pads. However, the storage and sunlight exposure of cloth pad data were not reported in all the articles, as mentioned earlier. 

Discussion

In our study, we estimated the pooled prevalence of menstrual hygiene practices variables in Indian tribal females. As per our knowledge, no meta-analysis has reported the pooled prevalence of use of sanitary pads among Indian tribal females yet. Kumar D. et al. noted menstrual hygiene aspects of tribal adolescent girls in Madhya Pradesh in a two-decade review where sanitary pad usage ranged from 53% to 83.78% [16]. However, result synthesis was not done by them due to heterogeneous data in a few studies. Similarly, Nandi P et al. also published a systematic review on menstrual hygiene practices in Indian adolescents [35]. 

The use of sanitary pads varies significantly in different tribal communities in India; most females prefer reusable old clothes due to cost factors, availability, and ease of disposal [16-25]. Contrary to our findings, tribal females residing outside India had a higher pooled prevalence of sanitary pad usage [36]. This difference may be due to community awareness, the limited availability of sanitary pads, and different beliefs and customs.

In our meta-analysis, there are few studies that report proper disposal of menstrual material after use. The used material was largely thrown indiscriminately, in village ponds, anywhere into a latrine, field, jungle, canal, or bin; flushed down a toilet, buried, wrapped in a newspaper, plastic, or paper; or left on the toilet floor [18,20,24]. A few other studies also reported the disposal of MHM in backyard waste for burning [34]. Kaur R et al. agree with our study’s finding of inappropriate disposal of menstrual material [37]. 

The knowledge of proper washing and drying of reusable clothes is limited to very few tribal women. Eijk AM et al. mentioned difficulties with changing, washing, and drying MHM in low- and middle-income countries, which is similar to our study findings [38]. We have found that tribal women prefer the use of old clothes to manage menstruation, and they dry their washed MHM under the other washed clothes so that no one can see them [32]. It was also noted that the same cloth is used over a long period to manage menstruation, which makes them vulnerable to reproductive and urinary infections [30]. The prevalence of sun-dried MHM was very low, as depicted in the results of this study, which were not yet calculated in any other meta-analysis.

We noted that menstruating females in some tribal communities did not brush their teeth or take a bath, slept on the floor, did not touch stored food items, and were restricted from eating certain foods during menstruation [20,21]. These findings corroborate previous studies conducted on menstrual hygiene among tribal females [37]. 

In the included studies, it was found that very few of the participants had complete knowledge about menstrual physiology before menarche, and some even believed that it was a curse of God. [16,18,33]. School absenteeism during menstrual days was also evident [20,33]. A similar perspective was reported by Eijk AM et al. [38].

Our meta-analysis was comprehensive and confirms that menstrual hygiene in tribal females is of great concern regarding their health upliftment.

Limitations

Many limitations came up while conducting this meta-analysis. Subgroup analysis was not performed in our study, as very few studies in the literature mentioned the data on reproductive-age females in community settings. Most of the included studies reported data on the adolescent age group. Hence, the findings cannot be considered representative of the community data on tribal females for menstrual hygiene. In addition, the study design of all selected articles was not uniform. Furthermore, the percentage prevalence of menstrual hygiene practices variables among the included studies was missing in a few.

## Conclusions

Menstruation is a significant marker of the initiation of reproductive life in females. The onset of menstruation (menarche) is celebrated in many communities. However, this study highlights that data on menstrual hygiene practices among Indian tribal females is inadequate, and even the available data shows that unsafe practices are prevalent in many areas. Community data from tribal females about their menstrual beliefs need to be collected proactively for planning future tribal health programs. Emphasis should be given to disposal and perineal hygiene practices in awareness campaigns in the respective areas. Further, an innovative socio-cultural perspective is necessary to dispel orthodox customs, false beliefs, and myths. 

## References

[1] Upashe SP, Tekelab T, Mekonnen J (2015). Assessment of knowledge and practice of menstrual hygiene among high school girls in Western Ethiopia. BMC Womens Health.

[2] Garg S, Anand T (2015). Menstruation related myths in India: strategies for combating it. J Family Med Prim Care.

[3] Belayneh Z, Mekuriaw B (2019). Knowledge and menstrual hygiene practice among adolescent school girls in southern Ethiopia: a cross-sectional study. BMC Public Health.

[4] Singh A, Chakrabarty M, Singh S, Chandra R, Chowdhury S, Singh A (2022). Menstrual hygiene practices among adolescent women in rural India: a cross-sectional study. BMC Public Health.

[5] Hennegan J, Nansubuga A, Akullo A, Smith C, Schwab KJ (2020). The Menstrual Practices Questionnaire (MPQ): development, elaboration, and implications for future research. Glob Health Action.

[6] Hulland KR, Chase RP, Caruso BA (2015). Sanitation, stress, and life stage: a systematic data collection study among women in Odisha, India. PLoS One.

[7] Paltasingh T, Paliwal G (2014). Tribal population in India: regional dimensions & imperatives. Jr Reg Devel Plan.

[8] Mathew A, Mathew A (2018). Traditional cultural practices, customs and beliefs of Paliya tribes inhabiting in Idukki district of Kerala. Jr Reg Dev Plan.

[9] Sharma S, Mehra D, Brusselaers N, Mehra S (2020). Menstrual hygiene preparedness among schools in India: a systematic review and meta-analysis of system-and policy-level actions. Int J Environ Res Public Health.

[10] Deshpande TN, Patil SS, Gharai SB, Patil SR, Durgawale PM (2018). Menstrual hygiene among adolescent girls - A study from urban slum area. J Family Med Prim Care.

[11] Robinson HJ, Barrington DJ (2021). Drivers of menstrual material disposal and washing practices: A systematic review. PLoS One.

[12] Ganguly L, Satpati L (2022). Sanitation condition and its association with menstrual hygiene of non-tribal and tribal communities in selected districts of West Bengal. Eco Env Con.

[13] Tzeghai GE, Ajayi FO, Miller KW, Imbescheid F, Sobel JD, Farage MA (2012). A feminine care clinical research program transforms women's lives. Glob J Health Sci.

[14] Achuthan K, Muthupalani S, Kolil VK, Bist A, Sreesuthan K, Sreedevi A (2021). A novel banana fiber pad for menstrual hygiene in India: a feasibility and acceptability study. BMC Womens Health.

[15] Barker TH, Stone JC, Sears K (2023). Revising the JBI quantitative critical appraisal tools to improve their applicability: an overview of methods and the development process. JBI Evid Synth.

[16] Kumar D, Vaiyam P, Thakur RS (2021). Management of menstrual hygiene, practices and perceptions among vulnerable Bharia women in Madhya Pradesh: A pilot survey. Jr Com Hea Man.

[17] Rose-Clarke K, Pradhan H, Rath S (2019). Adolescent girls' health, nutrition and wellbeing in rural eastern India: a descriptive, cross-sectional community-based study. BMC Public Health.

[18] Borkar SK, Borkar A, Shaikh MK, Mendhe H, Ambad R, Joshi A (2022). Study of menstrual hygiene practices among adolescent girls in a tribal area of Central India. Cureus.

[19] Sridhar D, Gauthami N (2017). Menstrual health status and cultural practices of tribal adolescent girls. Int J Community Med Public Health.

[20] Udayar SE, Kruthika K, Devi PV (2016). Menstrual hygiene practices among adolescent girls residing in tribal and social welfare hostel in Andhra Pradesh: a community based study. Ntl J Community Med.

[21] Shah SP, Nair R, Shah PP, Modi DK, Desai SA, Desai L (2013). Improving quality of life with new menstrual hygiene practices among adolescent tribal girls in rural Gujarat, India. Reprod Health Matters.

[22] Pal D, Chhillar J, Chhillar G, Chaturvedi M, Kataria U, Chhillar D (2022). Study to assess the knowledge and practice of menstrual hygiene among tribal women Sommaripeth at distt Betul, (M.P.). Int Jr Allied Med Sci Clin Res.

[23] Sawa T (2022). Tribal women of Jharkhand: a study of the menstrual health and sanitation. Int Jr Lat Res Hum Soc Sci.

[24] Kakwani J, Meena JK, Verma A, Dahiya N (2021). Emerging issues and barriers in access to menstrual hygiene management in a tribal district of India. Int J Community Med Pub Hea.

[25] Kakeri M, Patil SB, Waghmare R (2018). Knowledge and practice gap for menstrual hygiene among adolescent school girls of Tribal district of Maharashtra, India: a cross sectional study. Ind J Youth Adol Health.

[26] Ramya S (2020). A cross sectional study on menstrual hygiene practices among adolescent girls in tribal population in Tamil Nadu.

[27] Dey J, Mahapatra B (2020). Knowledge, practices and problem towards menstrual cycle with their socio economic status: a comparative study of tribal and non tribal female. Int J Com Med Pub Hea.

[28] Verma A, Patyal A, Mathur M, Mathur N, hiranij hiranij (2021). Prevalence of self-reported reproductive tract infections/sexually transmitted infections symptoms and treatment seeking behavior among the married tribal women in Udaipur, Rajasthan. Int J Reprod Contracept Obstet Gynecol.

[29] Das P, Baker KK, Dutta A (2015). Menstrual hygiene practices, WASH access and the risk of urogenital infection in women from Odisha, India. PLoS One.

[30] Mishra P (2020). Periods, perceptions and practice- a study of menstrual awareness among adolescent girls in a tribal district of Odisha, India. Ind Jr Pub Hea Res Dev.

[31] Mahapatra T (2023). Menstrual health and status of tribal adolescent girls of Balasore, Odisha. Inter Jr Sci Res.

[32] Birje S, Patil AD, Munne KR (2022). Enablers & challenges of tribal women & health system for implementation of screening of non-communicable diseases & common cancers: a mixed-methods study in Palghar district of Maharashtra, India. Indian J Med Res.

[33] Kumari S, Sood S, Davis S, Chaudhury S (2021). Knowledge and practices related to menstruation among tribal adolescent girls. Ind Psychiatry J.

[34] Vayeda M, Ghanghar V, Desai S (2021). Improving menstrual hygiene management among adolescent girls in tribal areas of Gujarat: an evaluation of an implementation model integrating the government service delivery system. Sex Reprod Health Matters.

[35] Nandi P (2022). Menstrual hygiene practices among adolescent girls and women in India—a systematic review. Int J Food Nutr Sci.

[36] Anbesu EW, Asgedom DK (2023). Menstrual hygiene practice and associated factors among adolescent girls in sub-Saharan Africa: a systematic review and meta-analysis. BMC Public Health.

[37] Kaur R, Kaur K, Kaur R (2018). Menstrual hygiene, management, and waste disposal: practices and challenges faced by girls/women of developing countries. J Environ Public Health.

[38] van Eijk AM, Jayasinghe N, Zulaika G, Mason L, Sivakami M, Unger HW, Phillips-Howard PA (2021). Exploring menstrual products: A systematic review and meta-analysis of reusable menstrual pads for public health internationally. PLoS One.

